# Barriers and Nursing Strategies in Oncology Care for LGBTQIA+ People: A Scoping Review

**DOI:** 10.3390/cancers17071146

**Published:** 2025-03-28

**Authors:** Gianluca Azzellino, Ernesto Aitella, Lia Ginaldi, Massimo De Martinis

**Affiliations:** 1 Department of Life, Health and Environmental Sciences, University of L’Aquila, 67100 L’Aquila, Italy; ernesto.aitella@graduate.univaq.it (E.A.); lia.ginaldi@univaq.it (L.G.); 2 Complex Operational Unit, Adriatic District Area, AUSL 04 Teramo, Italy; 3 Allergy and Clinical Immunology Unit, Center for the Diagnosis and Treatment of Osteoporosis, AUSL 04 Teramo, Italy; 4 Technical Group for the Coordination of Gender Medicine, Regione Abruzzo, Italy; 5 Long-Term Care Unit, “Maria SS. dello Splendore” Hospital, Giulianova, AUSL 04 Teramo, Italy; 6 UniCamillus-Saint Camillus International University of Health Sciences, 00131 Rome, Italy; 7 “Teramo hub” University of L’Aquila, 67100 L’Aquila, Italy

**Keywords:** LGBTQIA+ health, oncology nursing, nurse–patient communication, health disparities, inclusive care, nursing strategies, scoping review

## Abstract

LGBTQIA+ individuals with cancer face significant challenges in their interactions with nurses, which can negatively affect the quality of care, treatment adherence, and access to healthcare. Barriers such as implicit biases, discrimination, and inadequate communication skills contribute to these disparities, alongside a lack of nursing education on gender and sexual diversity. Despite the recognized role of nurses in reducing health inequalities, knowledge about effective strategies to overcome these barriers remains fragmented. This scoping review aims to identify and categorize the main barriers affecting the nurse–patient relationship in oncology and to explore evidence-based nursing interventions that promote equitable and inclusive care. A systematic literature search was conducted between January and February 2025 in PUBMED, SCOPUS, and Web of Science, including studies published in the last 10 years. Study management was performed using Zotero (version 6.0.30), and quality assessment was conducted with the JBI Critical Appraisal Tools. Two independent reviewers screened the studies, resolving discrepancies through discussion and consensus. This review follows the PRISMA-ScR checklist and the JBI scoping review methodology. The findings will provide insights into the primary barriers, highlight effective nursing interventions, and identify gaps in education and clinical guidelines, contributing to the development of more inclusive oncology care practices.

## Simple Summary

LGBTQIA+ individuals with cancer face unique challenges in healthcare settings, particularly in their interactions with nurses. Barriers such as implicit biases, discrimination, and inadequate communication skills can negatively impact the quality of care, treatment adherence, and access to healthcare. Despite the growing recognition of nurses’ role in reducing health disparities, there is still a lack of systematic knowledge regarding effective nursing strategies to support LGBTQIA+ patients in oncology. This scoping review aims to identify and categorize the main barriers affecting the nurse–patient relationship and explore evidence-based nursing interventions that improve care quality and equity. By mapping existing strategies and gaps, this study provides valuable insights for improving nursing education, developing inclusive clinical guidelines, and fostering a more equitable healthcare environment for LGBTQIA+ cancer patients.

## 1. Introduction

Lesbian, Gay, Bisexual, Transgender, Queer, Intersex, and Asexual (LGBTQIA+) individuals face numerous barriers to accessing oncology care, which negatively impacts the quality of care and health outcomes [1,2]. Recent studies demonstrate that this population is at a higher risk of developing certain cancers due to biological, social, and behavioral factors, including reduced access to screening programs, diagnostic delays, and minority stress [3,4,5,6,7]. Despite increasing awareness of these disparities, there is still a lack of systematic knowledge regarding effective nursing strategies to address them. The lack of specific training among healthcare providers and the absence of dedicated guidelines further hinder the provision of culturally competent oncology care [8,9]. One of the main obstacles to care for LGBTQIA+ individuals is the inadequate cultural competence of healthcare professionals, which results in experiences of discrimination and communication difficulties [10]. Furthermore, implementing inclusive nursing strategies—such as improving communication and creating more welcoming environments—can help reduce disparities in cancer care [11]. Barriers to care manifest at multiple levels:

Structural: The lack of data collection on gender identity and sexual orientation in clinical records prevents effective personalization of care [12]. The urgency of acquiring such data has been emphasized to ensure equity in cancer treatment and prevention [13].

Interpersonal: Many LGBTQIA+ patients report experiences of inadequate treatment, with stereotypical attitudes and communication difficulties with healthcare providers [14].

Individual: Fear of discrimination and the lack of a welcoming healthcare environment reduce adherence to cancer prevention and screening programs [8]. Additionally, the exclusion of LGBTQIA+ populations from clinical trials further limits evidence-based, inclusive approaches in oncology [15].

The nursing community plays a central role in improving oncology care for LGBTQIA+ patients. However, nursing education on these topics is often inadequate, with gaps in knowledge about the clinical and psychological specifics of this population [1]. Recent studies suggest that targeted training programs can improve nurses’ cultural competence, enhancing the quality of care provided and LGBTQIA+ patients’ adherence to oncology treatments [2,10]. Targeted interventions to improve inclusive communication and create more welcoming healthcare environments have proven effective in reducing inequalities in access to oncology care [11]. Additionally, missed nursing care, which encompasses the omission of essential nursing interventions, has emerged as a critical issue that further undermines the quality of oncology care, especially in settings where a high intention to leave among nursing staff exacerbates care disparities [16]. Addressing these challenges requires renewing the nursing profession to attract new talent while promoting an increasingly inclusive and diversity-conscious workforce [17]. In light of these issues, this scoping review aims to identify the main barriers affecting the nurse–patient relationship in oncology for LGBTQIA+ individuals, explore the most effective nursing interventions, and highlight gaps in education and clinical guidelines. The findings will contribute to the development of more inclusive oncology care practices, ultimately improving the quality of care and equity for LGBTQIA+ individuals.

## 2. Materials and Methods

This scoping review was conducted following the PRISMA-ScR checklist [18] and the JBI scoping review methodology [19]. Additionally, the review followed the updated methodological guidance for scoping reviews proposed by Peters et al. [20], ensuring a rigorous and transparent synthesis of the available evidence [20] This scoping review was not registered in any database, as registration is not a mandatory requirement for this type of study. However, to ensure transparency and reproducibility, all methodological steps were rigorously followed, including the definition of eligibility criteria, search strategy, data extraction, and synthesis. The research questions that guided this review were as follows: What are the main barriers faced by LGBTQIA+ cancer patients? What nursing interventions can promote more inclusive and equitable care? To answer these questions, the PCC framework was applied:

- Population: LGBTQIA+ cancer patients.
- Concept: Barriers faced by LGBTQIA+ patients in oncology care and nursing interventions to improve care quality and equity.
- Context: Oncology care settings, including hospitals, outpatient clinics, and palliative care services.

### 2.1. Data Sources and Search Strategy

The search was conducted using the PubMed, Scopus, and Web of Science electronic databases. No additional search strategies were employed. Table 1 shows the search strings used in each database.

### 2.2. Data Extraction

Using JBI scoping review methodology, data were extracted from the articles included in this review using a results extraction table [21]. Data were extracted using a standardized form that included the following: author/years, main theme, geographical context, study design and methods, population and sample characteristics, key findings, and research gaps.

### 2.3. Study Selection

Studies were included if they met the following criteria:

- Focused on the experience of LGBTQIA+ patients in oncology care;
- Addressed barriers to care, treatment adherence, or communication with healthcare professionals;
- Discussed interventions, educational programs, or strategies implemented in oncology care to improve inclusivity;
- Published in English or Italian and included studies from the last 10 years (2010–2024).

Exclusion criteria included the following:

- Studies that did not focus on the LGBTQIA+ population or oncology care;
- Articles without full-text access or that were purely theoretical with no empirical data.

### 2.4. Screening Process

**Figure 1:**
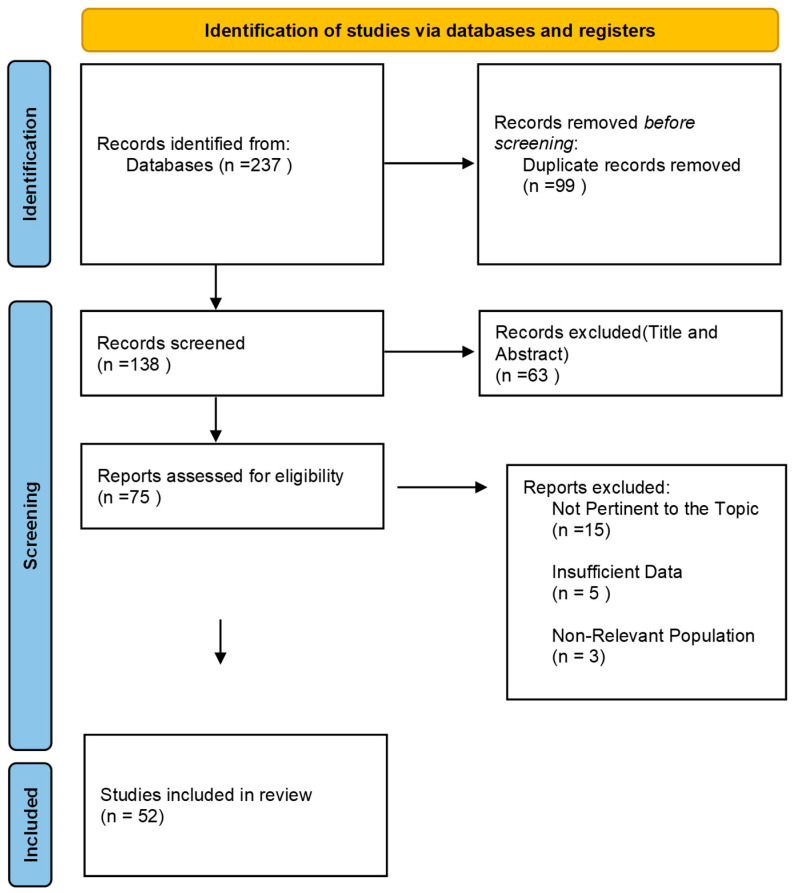
PRISMA 2020 flow diagram illustrating the study selection process. The databases searched were PubMed, Scopus, and Web of Science.

The study selection process was carried out in two stages to ensure that only relevant studies were included in this review. In the first stage, two independent reviewers screened the titles and abstracts of the studies identified through the search strategy to assess their eligibility based on the inclusion and exclusion criteria. In the second stage, the full texts of the selected studies were reviewed to confirm their inclusion. The study selection process is illustrated in the PRISMA 2020 flow diagram (PRISMA Statement, 2021) (Figure 1).

### 2.5. Quality Assessment and Risk of Bias

In this review, an assessment of risk of bias was not performed, as scoping reviews typically do not require an in-depth quality evaluation of the included studies. The main objective of this review was to synthesize and map the available evidence rather than critically appraise each individual study. This approach is consistent with the methodological guidelines for scoping reviews, as outlined in the JBI Manual for Evidence Synthesis and the PRISMA-ScR.

### 2.6. Data Synthesis

The synthesis of results followed the approach outlined in the JBI Manual for Evidence Synthesis (2020) for scoping reviews. The extracted data were organized into a descriptive synthesis without critical appraisal of methodological quality, as recommended for this type of review. The synthesis was structured in three key phases: Identification of key themes: After data extraction, two independent reviewers analyzed the content to identify barriers, nursing strategies, and broader implications for LGBTQIA+ oncology care. Grouping of results: Data were categorized into thematic groups based on recurring topics across the included studies. Narrative synthesis: The results were qualitatively described and synthesized while maintaining fidelity to the original data, providing a comprehensive overview of the available evidence. The results were classified into three main categories: barriers faced by LGBTQIA+ cancer patients in oncology care, including limited cultural competence among healthcare providers, implicit biases, and lack of inclusive policies; nursing interventions to improve inclusivity and equity in oncology care, such as targeted training programs, inclusive communication strategies, and environmental modifications to enhance patient comfort; and implications for LGBTQIA+ patients and healthcare providers in oncology settings, emphasizing the need for systemic changes in education, policy, and clinical practice. Artificial Intelligence (AI), specifically ChatGPT-4o (OpenAI, San Francisco, CA, USA), was used exclusively to support the refinement of the language and style of the manuscript. All scientific decisions and methodological steps were carried out solely by the authors.

## 3. Results

### 3.1. Overview of Included Studies

**Figure 2:**
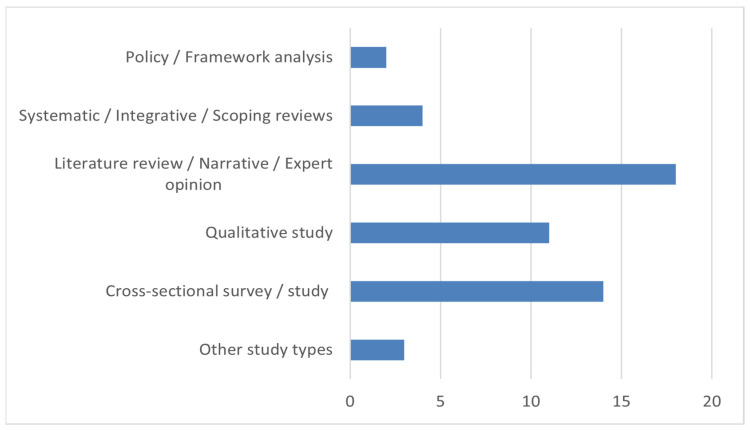
Distribution of study types included in this review.

A total of 237 studies were identified in the initial stages, of which 52 met the inclusion criteria (Table 2). Most of the studies included were cross-sectional surveys, followed by literature reviews, qualitative studies, and narrative reviews. Other methodological approaches noted include mixed-method studies, systematic reviews, and expert opinion-based analyses (Figure 2).

### 3.2. Main Results

**Figure 3:**
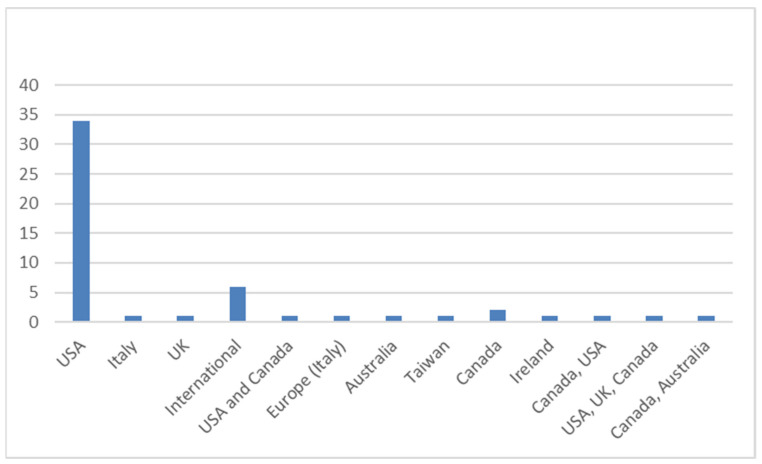
Geographical distribution of included studies.

Figure 3 shows the geographical distribution of the included studies, highlighting that most were conducted in the USA.

The analysis of the literature revealed six key thematic areas related to the barriers and challenges faced by LGBTQIA+ patients in oncology care and the critical role of nursing interventions in promoting more inclusive and equitable care.

- **Cultural Competence and Microaggressions**

Several studies indicate that inadequate cultural competence among healthcare providers is a significant barrier to effective cancer care for LGBTQIA+ patients. Oncology professionals, in particular, reported feeling unprepared to manage the specific needs of this population, which can lead to misgendering, outdated terminology use, and avoidance of discussions about sexual orientation and gender identity [22,49]. These microaggressions negatively impact patient trust and may discourage LGBTQIA+ individuals from seeking timely cancer care [26]. While microaggressions such as misgendering, outdated terminology, and avoidance of discussions about sexual orientation and gender identity negatively impact LGBTQIA+ patients’ trust in healthcare providers [26], it is important to distinguish these issues from necessary clinical decisions based on biological sex. In oncology, drug dosages, risk assessments, and treatment plans are often determined by biological factors such as hormone levels and anatomical structures rather than gender identity. This can create a potential dilemma between the patient’s perspective and the oncologist’s medical approach, highlighting the need for improved communication strategies to ensure both clinical accuracy and culturally competent care. To address these issues, some studies have highlighted the importance of structured training programs aimed at improving healthcare providers’ cultural competence. Evidence suggests that such programs enhance communication and reduce implicit biases, ultimately improving healthcare experiences for LGBTQIA+ individuals [10].

- **Disparities in cancer screening**

Scoping reviews have consistently shown that LGBTQIA+ individuals have lower rates of adherence to cancer screening programs due to a combination of discrimination, fear of stigma, and lack of culturally competent healthcare providers [23]. Transgender people, in particular, have been found to be disadvantaged by the lack of specific guidelines for cancer screening [24]. Furthermore, standard screening protocols do not adequately consider the needs of transgender and non-binary individuals, leading to diagnostic delays and poorer cancer prognoses [61]. These findings underscore the urgent need for tailored screening recommendations and educational initiatives to ensure equitable cancer detection and prevention.

- **Barriers to access to care**

Transgender patients face significant barriers, including denial of gender-affirming care, stigma, and difficulty with health insurance [24]. Additionally, this population has high rates of mental health disorders, including depression and suicide risk, which negatively impact access to cancer services [45].

- **Gaps in oncologists’ knowledge**

Several studies revealed a lack of knowledge among oncologists regarding LGBTQIA+ health, particularly in relation to the specific medical and psychosocial needs of transgender patients [26]. Despite recognizing the importance of LGBTQIA+ sensitivity training, many oncologists report a lack of structured programs on this topic [1]. However, research indicates that participation in targeted training programs significantly improves cultural competence, communication skills, and overall care quality for LGBTQIA+ cancer patients [10]. These findings reinforce the need to integrate LGBTQIA+-inclusive curricula into oncology education and continuing medical training.

- **Missed Nursing Care and Critical Issues in Palliative Care**

The omission of essential nursing interventions has emerged as a critical issue, exacerbated by the high turnover rate among healthcare workers [16]. LGBTQIA+ patients in palliative care have faced increased social isolation and poor inclusion in end-of-life policies [28,32], highlighting the need for specific protocols to ensure more inclusive care.

- **Sexual and Reproductive Health in LGBTQIA+ Cancer Patients**

Discussions about sexual and reproductive health have often been neglected in LGBT+ oncology. Many healthcare providers do not receive training on these issues, leading to ineffective communication and suboptimal management of these aspects of care [27]. Transgender and non-binary people, in particular, face difficulties in accessing genetic counseling and fertility care in oncology [38]. Studies emphasize that improving social support networks and access to LGBTQIA+-friendly reproductive services significantly enhances psychological well-being and patient satisfaction [41].

### 3.3. Research Gaps

Despite the growing attention on cancer health among LGBTQIA+ people, several research gaps persist [30,31,33,34,36,39,43].

**Limited Evidence on Cancer Prevention and Screening for Transgender Individuals**

Current cancer screening guidelines do not address the specific needs of transgender individuals, which contributes to lower adherence to screenings and increased cancer risk [23,24]. However, studies indicate that additional barriers, such as fear of discrimination and past negative experiences with healthcare providers, may play an even more significant role in discouraging participation in preventive programs [23]. The lack of clear screening protocols also leads to uncertainty among healthcare providers, resulting in inconsistent recommendations and limited proactive engagement in preventive care [23,61]. Addressing these barriers requires both the development of specific guidelines and the implementation of inclusive healthcare policies to foster trust and engagement in cancer prevention.

**Lack of Standardized Training Programs**

The need for structured, evidence-based educational interventions to improve cultural competence in oncology care remains unresolved. Studies indicate that LGBTQIA+ health training among healthcare professionals is limited and often not mandatory, leading to gaps in the management of LGBTQIA+ patients [1,10,26]. Clinical challenges have already been reported, particularly in the management of breast cancer among transgender individuals, where clinicians often lack specific training and formal guidance [35]. Oncologists, in particular, receive little training on the specific needs of transgender patients and inclusive communication strategies [37,50].

**Absence of National and Institutional Policies Supporting Inclusion**

Many studies have highlighted the lack of inclusive healthcare policies and regulations to ensure non-discriminatory healthcare environments for LGBTQIA+ patients [28,29,50]. The absence of specific policies results in inadequate protection against discrimination and insufficient mandatory training for healthcare professionals.

**Insufficient Data on Sexual Orientation and Gender Identity (SOGI)**

The lack of structured data collection on sexual orientation and gender identity (SOGI) in clinical records and national databases limits research progress and the development of targeted healthcare policies [13,40]. While some challenges in cancer screening for transgender individuals have been identified—such as the lack of tailored screening guidelines and barriers to accessing gender-affirming care—further research is needed to establish standardized recommendations and assess the long-term impact of current screening disparities on LGBTQIA+ cancer outcomes.

**Barriers to Palliative and End-of-Life Care**

The specific needs of LGBTQIA+ patients in palliative care remain under-researched, particularly concerning the inclusion of chosen family and psychological support [28,32,42]. Studies indicate that LGBTQIA+ individuals often avoid palliative care due to fear of discrimination and a lack of healthcare providers trained in their social and familial realities.

**Lack of Training on Inclusive Communication**

The integration of inclusive communication strategies into medical and nursing education is necessary to improve relationships between patients and healthcare providers and to increase trust in the healthcare system [10,22,26]. Studies show that training on inclusive communication can reduce LGBTQIA+ patients’ fear of discrimination and improve adherence to oncology treatments [11,60].

## 4. Discussion

The results of this scoping review highlight that LGBTQIA+ individuals face significant barriers in accessing oncology care, a problem already documented in previous studies [23,29]. To achieve better health for all individuals, we need to move quickly to expand research on social determinants of health (SDOH) and other important factors. All health professionals are becoming increasingly attentive to social needs at the individual level, trying to adapt care to meet those needs while taking into account the local community context and connecting patients with the resources needed to care for them [62]. Unfortunately, there is still a lot of progress to be made to achieve these goals in a political/cultural context that often does not help. Nursing cannot be separated from social justice and professionals must work to correct unjust systems and processes, promote social change, and achieve health equity. Social justice allows for equitable and inclusive universal access to healthcare and is recognized by the International Council of Nurses (ICN) (2021) as an ethical concept that should translate into a “way of being and responding to people in the context of everyday nursing practice” to remove oppressive social structures and inequalities [63]. Sex and gender minorities represent populations with specific care needs, often unrecognized or underestimated, which, in particular, in the case of oncological care, increase the difficulties and suffering to be faced.

Of particular importance are the psychosocial aspects often already significant and present in these population groups. The role of the nurse in these contexts is delicate and demanding, often presenting problems that one is not sufficiently prepared to face. Despite this, nurses are probably the most suitable, as they are the closest to the patient and thus could possess the keys to give the most appropriate answers. The lack of cultural competence among healthcare providers emerges as one of the main obstacles, with many professionals reporting insufficient preparation in managing the specific needs of this population [1,26]. This translates into microaggressions, misgendering, and communication difficulties, which undermine the trust of LGBTQIA+ patients in the healthcare system [22,49]. Another key finding concerns the low adherence to cancer screening programs among LGBTQIA+ individuals, particularly transgender people, due to the absence of specific guidelines and the fear of stigma [3,24]. Access to care is further hindered by the lack of insurance coverage for gender-affirming treatments and the high prevalence of mental health disorders among transgender individuals, increasing the risk of delaying or avoiding contact with the healthcare system [35,61,64]. Moreover, the lack of systematic data collection on sexual orientation and gender identity in clinical records limits the ability to develop targeted interventions, obstructing the improvement of oncology care for this population [40,65,66].

### 4.1. Implications for Clinical Practice

The results of this review confirm the need for interventions to improve the inclusivity of oncology care for LGBTQIA+ individuals. The key recommended actions are as follows.

**Enhancing Healthcare Providers’ Training on Cultural Competence**

Previous studies have demonstrated that specific training programs can improve the quality of care and communication between LGBTQIA+ patients and healthcare professionals [2,10]. However, training on LGBTQIA+ health remains optional and fragmented [37,50], highlighting the need for mandatory curricula for all healthcare professionals [46,47,67,68,69]. One critical gap in oncology education is the lack of structured training on post-surgical care for transgender and non-binary patients, including mastectomy, breast augmentation, orchiectomy, hysterectomy, and other gender-affirming procedures. These interventions require tailored follow-up, yet many healthcare providers report insufficient knowledge in managing pain, wound care, and long-term complications such as fibrosis, lymphedema, and hormone-related cancer risks [2,37,50]. Patel et al. emphasize the need for inclusive cancer care strategies that consider these post-surgical challenges [2]. Additionally, Roznovjak et al. highlight the absence of standardized screening guidelines for transgender individuals, impacting post-surgical monitoring and long-term oncology outcomes [37]. Kano et al. advocate for the integration of LGBTQIA+-focused training programs into oncology education to address these knowledge gaps [50].

**Developing Oncology Screening Guidelines for Transgender Individuals**

Current cancer screening guidelines are primarily based on cisnormative categories, excluding the specific needs of transgender individuals undergoing hormone therapy or post-surgical interventions [35,48,52,53,54,61]. Integrating specific protocols is essential to ensure equitable access to cancer prevention programs [55,56,57,58,59].

**Integrating the Collection of Sexual Orientation and Gender Identity (SOGI) Data**

Systematic inclusion of SOGI data in healthcare records would enable better personalization of care and facilitate research on health disparities [12,13]. Implementing these systems requires adequate staff training to ensure the correct and respectful use of collected information [55]. However, some LGBTQIA+ patients may be reluctant to disclose this information due to concerns about privacy, potential discrimination, or lack of trust in healthcare systems. Studies indicate that these concerns can lead to the underreporting of SOGI data, potentially limiting the effectiveness of personalized healthcare strategies [1]. Therefore, policies on SOGI data collection should ensure that disclosure remains voluntary, confidential, and patient-centered, promoting trust and inclusivity.

**Addressing Challenges in Palliative Care and Sexual Health Management**

LGBTQIA+ individuals in palliative care often experience social isolation and discrimination, with insufficient recognition of “chosen family” in care pathways [28,32,42]. Additionally, sexual and reproductive health is rarely discussed with LGBTQIA+ patients, creating an additional care gap that must be addressed through dedicated training programs [25,41,51,60].

### 4.2. Limitations of the Study

This scoping review has some limitations. The selection of studies was based on indexed sources, potentially excluding unpublished data or policy documents. Additionally, the descriptive nature of this review does not allow for quantifying the effectiveness of the analyzed interventions.

### 4.3. Future Perspectives

To address current gaps, future research should focus on the following:

- Developing and evaluating training programs for healthcare providers, with an emphasis on inclusive communication and the specific needs of transgender and non-binary individuals.
- Analyzing long-term oncological outcomes in LGBTQIA+ populations, with particular attention to the influence of hormone therapies on cancer risks.
- Assessing the effectiveness of inclusive healthcare policies in oncology care to determine which strategies can effectively reduce disparities in access to treatment.

## 5. Conclusions

This scoping review highlights the need for a paradigm shift in oncology care for LGBTQIA+ individuals. Mandatory training for healthcare providers, the adoption of specific guidelines for transgender individuals, and the integration of SOGI data into health records are essential steps to reduce existing disparities. Investing in more inclusive healthcare policies and targeted research will be crucial to ensuring equity in the access to and quality of oncology care for the LGBTQIA+ population.

## Figures and Tables

**Table 1:** Search strategies used in each database.

Database	Search String	Articles Found
PUBMED	(nurse* OR “nursing staff” OR “healthcare provider*” OR “oncology nurse*”) AND (LGBT OR LGBTQ OR “sexual minorities” OR “gender minority” OR “gender diverse” OR transgender OR “non-binary” OR homosexual OR bisexual) AND (barrier* OR challenge* OR obstacle* OR “communication barrier*” OR “organizational barrier*” OR “educational barrier*” OR “training barrier*”) AND (cancer OR oncology OR “cancer care” OR “oncologic care” OR “cancer treatment”)	49
	(“evidence-based practice” OR “best practice*” OR intervention* OR “nursing strategy*” OR “nursing intervention*” OR “culturally competent care”) AND (nurse* OR “oncology nurse*” OR “healthcare provider*”) AND (LGBT OR LGBTQ OR “gender minority” OR “sexual minority”) AND (cancer OR oncology OR “oncologic patient*” OR “cancer care”)	28
SCOPUS	(TITLE-ABS-KEY (nurse* OR “nursing staff” OR “healthcare provider*” OR “oncology nurse*”)) AND (TITLE-ABS-KEY (LGBT OR LGBTQ OR “sexual minorities” OR “gender minority” OR “gender diverse” OR transgender OR “non-binary” OR homosexual OR bisexual)) AND (TITLE-ABS-KEY (barrier* OR challenge* OR obstacle* OR “communication barrier*” OR “organizational barrier*” OR “educational barrier*” OR “training barrier*”)) AND (TITLE-ABS-KEY (cancer OR oncology OR “cancer care” OR “oncologic care” OR “cancer treatment”))	55
	(TITLE-ABS-KEY (“evidence-based practice” OR “best practice*” OR intervention* OR “nursing strategy*” OR “nursing intervention*” OR “culturally competent care”)) AND (TITLE-ABS-KEY (nurse* OR “oncology nurse*” OR “healthcare provider*”)) AND (TITLE-ABS-KEY (LGBT OR LGBTQ OR “gender minority” OR “sexual minority”)) AND (TITLE-ABS-KEY (cancer OR oncology OR “oncologic patient*” OR “cancer care”))	21
WEB OF SCIENCE	(nurse* OR “nursing staff” OR “healthcare provider*” OR “oncology nurse*”) AND (LGBT OR LGBTQ OR “sexual minorities” OR “gender minority” OR “gender diverse” OR “transgender” OR “non-binary” OR “homosexual” OR “bisexual”) AND (barrier* OR challenge* OR obstacle* OR “communication barrier*” OR “organizational barrier*” OR “educational barrier*” OR “training barrier*”) AND (cancer OR oncology OR “cancer care” OR “oncologic care” OR “cancer treatment”)	65
	(“evidence-based practice” OR “best practice*” OR intervention* OR “nursing strategy*” OR “nursing intervention*” OR “culturally competent care”) AND (nurse* OR “oncology nurse*” OR “healthcare provider*”) AND (LGBT OR LGBTQ OR “gender minority” OR “sexual minority”) AND (cancer OR oncology OR “oncologic patient*” OR “cancer care”)	19

**Table 2:** Characteristics of included studies (=52).

Author/ Year	Main Theme	Geographical Context	Study Type	Sample/Population	Key Findings	Research Gaps
Patterson et al. [22]	Cultural competency and microaggressions in the provision of care to LGBT patients	USA	Cross-sectional study with mixed methods (survey + qualitative interviews)	5 healthcare providers (nurses and physicians); 6 in-depth interviews	- Only 54.1% felt competent in LGBT patient care. - Oncology providers felt less competent than primary care providers. - Providers described microaggressions (misgendering, use of outdated terms, avoidance of discussions on sexual orientation/gender identity).	- Lack of training addressing personal biases and implicit discrimination. - Need for educational programs that go beyond knowledge to include self-awareness training.
Ceres et al. [23]	Cancer screening considerations and uptake for LGBT persons	USA	Review of literature and national guidelines	Review of published studies and cancer screening recommendations for LGBT individuals	- LGBT individuals have increased cancer risks but lower screening rates due to discrimination, lack of cultural competence among healthcare providers, and fear of stigma. - Gay and bisexual men have higher healthcare utilization but may face discrimination in screening contexts. - Lack of specific cancer screening guidelines tailored for LGBT individuals.	- Limited data on cancer screening uptake among transgender individuals. - Insufficient physician training in LGBT health needs. - Lack of national databases collecting sexual orientation and gender identity (SOGI) information, limiting research and policy development.
Rowe et al. [24]	Barriers to healthcare access for transgender patients and strategies to improve care	USA	Narrative review	Review of studies and healthcare reports on transgender patient experiences	- Transgender individuals face discrimination, stigma, and lack of cultural competence from healthcare providers. - Insurance often denies coverage for gender-affirming care. - High rates of mental health disorders, including anxiety, depression, and suicide attempts. - Barriers in routine screenings for cancer due to provider biases and lack of clinical guidelines.	- Need for standardized education for healthcare providers on transgender-specific health needs. - Lack of policies ensuring inclusive and non-discriminatory care environments.
Baldwin et al. [25]	Health and identity-related interactions between lesbian, bisexual, queer, and pansexual women and their healthcare providers	USA	Mixed-method study (survey with qualitative and quantitative analysis)	Online survey of lesbian, bisexual, queer, and pansexual women across the USA	- Disclosure of sexual identity varies by identity group, with bisexual and pansexual women being less likely to disclose compared to lesbians. - Healthcare interactions and motivations for disclosure impact patient experience and care quality.	- Limited understanding of how different LGBTQ+ subgroups experience healthcare interactions. - Lack of specific training for healthcare providers on engaging with diverse sexual identities.
Banerjee et al. [26]	Knowledge, beliefs, and communication behavior of oncology healthcare providers regarding LGBT patient care	USA	Cross-sectional survey study	1253 oncology healthcare providers (HCPs) including physicians, advanced practice professionals, and nurses	- Only 5% of HCPs answered all knowledge questions about LGBT health correctly. - Higher knowledge was linked to better communication behaviors, especially with transgender patients. - 80% of HCPs had treated an LGB patient, but only 28% had treated a transgender patient. - Most HCPs believed LGBT sensitivity training would improve communication.	- Limited training on LGBT health among oncology providers. - Lack of institutional policies ensuring culturally competent care. - Need for structured, evidence-based LGBT sensitivity programs in oncology.
Barcellini et al. [27]	Awareness and attitudes of oncology healthcare providers towards sexual health in women and sexual-gender minority (SGM) cancer patients	Italy	Cross-sectional survey	184 Italian oncology clinicians (gynecologists, medical oncologists, radiation oncologists)	- Gender identity and sexual orientation were not routinely assessed. - 39.7% of clinicians reported difficulty in addressing sexual health topics. - Radiation oncologists were more likely to refer patients for sexual dysfunction management.	- Lack of standardized training programs on sexual health for oncologists. - Insufficient integration of sexual health discussions in routine oncology care. Need for specific guidelines for managing sexual dysfunction in SGM patients.
Barret et al. [28]	Barriers and strategies in providing palliative care to LGBTQ+ patients	USA	Narrative review	Analysis of barriers faced by LGBTQ+ individuals in palliative care settings	- LGBTQ+ patients face stigma, discrimination, and lack of trust in healthcare professionals. - Absence of inclusive healthcare policies addressing the needs of LGBTQ+ patients. - Inadequate training among healthcare providers on LGBTQ+ cultural competency. - LGBTQ+ patients experience higher levels of isolation and lack of family support, impacting their end-of-life care.	- Need for evidence-based training programs for healthcare professionals. - Limited data on the impact of inclusive policies on patient outcomes in palliative care.
Boehmer (2018) [29]	Barriers to cancer care for LGBT populations	USA	Literature review	Analysis of published studies on LGBT disparities in oncology care	- LGBT individuals face systemic barriers in cancer care, including lack of health insurance, economic disparities, and limited access to screening. - There are differences in cancer screening rates by sexual orientation, with lower cervical cancer screening among lesbian and bisexual women. - LGBT patients are more likely to experience discrimination and avoid seeking care due to past negative healthcare experiences.	- Lack of research on cancer survivorship experiences among LGBT individuals. - Limited knowledge of how social determinants of health affect cancer outcomes in LGBT populations.
Chandler (2020) [30]	Inclusion of LGBT+ patients in nursing services, with a focus on stoma care	UK	Observational study	Analysis of barriers faced by LGBT+ patients in stoma care and nursing interventions	- LGBT+ patients face discrimination and stigma in healthcare settings, leading to emotional distress and social isolation. - Lack of cultural competence among nurses affects the quality of care for LGBT+ patients. - Inclusive policies and training programs are needed to ensure sensitive and respectful nursing care. - Creating a welcoming environment improves patient trust and adherence to treatment.	- Limited research on the specific needs of LGBT+ individuals with stomas. - Need for structured training programs on LGBT+ inclusion in nursing education. - Lack of standardized guidelines for inclusive stoma care in LGBT+ populations.
Chan et al. (2023) [31]	Needs and experiences of cancer care in LGBTQ+ patients	International	Systematic review	Analysis of existing studies on LGBTQ+ patients in oncology care	- LGBTQ+ patients face discrimination and stigma, leading to healthcare avoidance and lack of disclosure of sexual orientation/gender identity. - Many healthcare providers lack cultural competence, negatively affecting patient experiences. - Psychological support is often inadequate and not tailored to LGBTQ+ needs. - Inclusive policies and professional training are necessary to improve cancer care.	- Limited research on intersectionality (e.g., race, socioeconomic status) in LGBTQ+ oncology care. - Need for standardized guidelines for inclusive cancer treatment. - Lack of large-scale studies on long-term cancer outcomes for LGBTQ+ individuals.
Cloyes et al. (2018) [32]	Palliative and end-of-life care for LGBT+ cancer patients and their caregivers)	USA	Literature review and clinical guidelines synthesis	Analysis of research on LGBT+ cancer patients in palliative care	- LGBT+ patients face unique challenges during care transitions, including provider communication barriers and lack of recognition of chosen family. - Many LGBT+ patients distrust healthcare providers due to past discrimination. - Spiritual needs of LGBT+ patients are often overlooked in palliative care. - Inclusion of LGBT+ cultural competency in nursing education improves care quality.	- Limited research on LGBT+ patients’ experiences in hospice and end-of-life care. - Need for standardized training programs for oncology and palliative care providers. - Lack of policies ensuring inclusive, affirming environments for LGBT+ patients and caregivers.
Kempinski (2024) [33]	Creating LGBTQIA+-inclusive healthcare as a supportive care strategy in oncology	USA	Clinical review and expert recommendations	Analysis of disparities and best practices for culturally competent oncology care	- LGBTQIA+ individuals face significant health disparities, including lower screening rates and higher cancer risks. - Stigma and discrimination contribute to healthcare avoidance and poor patient–provider communication. - Oncology nurses play a critical role in creating inclusive, affirming healthcare environments. - Cultural competency training is essential to reduce barriers and improve care.	- Limited research on the long-term impact of culturally competent interventions in oncology. - Need for standardized oncology guidelines specific to LGBTQIA+ populations. - Lack of large-scale studies on the effects of inclusive healthcare policies in oncology settings.
Daniels et al. (2023) [34]	Impact of prostate cancer on gay and bisexual men and their relationships	USA and Canada	Qualitative study (focus groups)	12 gay and bisexual men with prostate cancer	- Prostate cancer diagnosis and treatment affect sexual function, identity, and relationship dynamics among GB men. - Communication barriers with healthcare providers limit discussions on sexual health and treatment side effects. - Many participants reported avoiding disclosure of sexual orientation to providers due to fear of stigma. - GB men often felt isolated and lacked adequate support systems post-treatment.	- Need for tailored interventions addressing sexual health concerns in GB prostate cancer patients. - Limited healthcare provider training on GB-specific cancer survivorship challenges. - Lack of research on long-term mental health outcomes for GB prostate cancer survivors.
Fehl et al. (2019) [35]	Breast cancer in the transgender population	USA	Case study and literature review	Case study of a 41-year-old transgender male with breast cancer + review of literature on breast cancer in transgender individuals	- Transgender individuals face unique challenges in breast cancer diagnosis and treatment, including misgendering, lack of gender-affirming care, and systemic barriers. - Testosterone therapy and mastectomy influence breast cancer risk in transgender men, but data on long-term effects are limited. - Oncology providers often lack training in transgender health needs.	- Need for clear, evidence-based breast cancer screening guidelines for transgender individuals. - Limited research on the impact of long-term testosterone therapy on breast cancer risk. - Insufficient provider training on transgender-specific oncology care.
Gannon et al. (2022) [36]	Understanding the influences of healthcare provider (HCP)-patient interactions in cancer care for LGBTQ+ children and young people	International	Qualitative study	Analysis of communication barriers and healthcare interactions in pediatric oncology for LGBTQ+ youth	- LGBTQ+ young patients face difficulties in disclosing their sexual orientation and gender identity due to fear of discrimination. - Healthcare providers’ cultural competence plays a key role in creating an inclusive and safe environment. - Open and empathetic communication improves the psychological well-being and treatment adherence of LGBTQ+ patients. - Lack of proactive discussions on sexual and gender identity leads to patient alienation.	- Need for structured training programs on LGBTQ+ inclusivity for healthcare providers. - Limited data on long-term outcomes of LGBTQ+ pediatric cancer patients. - Insufficient policies ensuring LGBTQ+ inclusivity in pediatric oncology settings.
Gentile et al. (2020) [37]	Clinicians’ experience, self-perceived knowledge, and attitudes toward LGBTQ+ health topics	USA	Cross-sectional survey	880 physicians and advanced practice providers	- Only 6–10% of clinicians reported sophisticated knowledge of LGBTQ+ health topics. - Positive attitudes were common, but knowledge gaps were significant, especially on transgender health. - Clinical experience was not associated with greater knowledge or attitudes toward LGBTQ+ health issues. - 54% of clinicians preferred online education for LGBTQ+ topics.	- Limited LGBTQ+ medical education across different experience levels. - Need for standardized LGBTQ+ cultural competence training in medical curricula. - Insufficient research on the direct impact of knowledge gaps on clinical care outcomes for LGBTQ+ patients.
Mahon (2023) [38]	Cancer genetic counseling and clinical care for transgender and non-binary individuals	USA	Clinical review and expert recommendations	Analysis of challenges and strategies for inclusive genetic counseling in oncology for TG/NB individuals	- TG/NB individuals may face barriers to accessing genetic counseling due to concerns about marginalization and lack of understanding. - Cancer genetic testing results can influence gender-affirming care decisions, including hormone therapy and surgery. - Inclusive care must be integrated at all stages, from scheduling to post-test counseling. - Nurses play a critical role in providing psychosocial support and identifying/removing barriers to care.	- Limited research on the impact of genetic counseling on cancer prevention in TG/NB populations. - Need for standardized, inclusive genetic counseling guidelines. - Insufficient provider education on integrating gender-affirming care with genetic risk assessment.
Gibson et al. (2017) [39]	Cancer care disparities in LGBTQ+ populations	International	Literature review	Analysis of barriers and interventions in oncology care for LGBTQ+ patients	- LGBTQ+ individuals face significant healthcare disparities in oncology, including barriers to access, delayed diagnoses, and inadequate treatment. - Fear of discrimination leads to avoidance of medical care, negatively impacting health outcomes. - Healthcare professionals lack cultural competence in addressing LGBTQ+ cancer care needs. - Training programs and inclusive policies are essential to reducing disparities and improving care quality.	- Limited research on long-term cancer outcomes in LGBTQ+ patients. - Need for tailored interventions in oncology that address specific LGBTQ+ needs. - Insufficient data on the impact of inclusive healthcare policies in oncology settings.
Ginaldi & De Martinis (2024) [40]	Interventions targeting LGBTQIA+ populations to advance health equity	Europe (Italy)	Policy analysis and expert commentary	Review of healthcare disparities and interventions for LGBTQIA+ individuals	- LGBTQIA+ individuals face significant healthcare disparities due to discrimination, lack of provider knowledge, and limited data collection. - Structural and systemic inequalities lead to reduced access to healthcare, lower screening rates, and poorer outcomes. - Collecting accurate sexual orientation and gender identity (SOGI) data is essential for improving care. - Healthcare providers require comprehensive LGBTQIA+ cultural competency training.	- Lack of standardized frameworks for integrating LGBTQIA+ health needs into universal healthcare systems. - Limited research on chronic disease management in LGBTQIA+ populations. - Need for global initiatives to promote inclusive policies beyond high-income countries.
Gorman et al. (2024) [41]	Sexual and reproductive healthcare experiences of transgender and gender-diverse (TGD) cancer survivors	USA	Qualitative study	17 TGD cancer survivors and 5 co-survivors (support persons)	- Cancer treatment’s impact on sexual health is often overlooked in discussions with TGD patients. - Fertility-related information is frequently inadequate or based on gendered assumptions. - TGD individuals face exclusion in healthcare settings due to gendered language and lack of supportive services. - High financial burden limits access to cancer care and fertility preservation. - Strong social support networks improve TGD survivors’ experiences and outcomes.	- Need for gender-affirming healthcare providers and environments. - Limited research on TGD cancer survivorship and reproductive health. - Lack of standardized SRH guidelines tailored for TGD cancer patients. - Insufficient provider training on inclusive sexual and reproductive healthcare.
Haviland et al. (2020) [42]	Barriers and facilitators to cancer screening among LGBTQ+ individuals	USA	Integrative review	Review of 12 studies on cancer screening behaviors in LGBTQ+ populations	- LGBTQ+ individuals are less likely to undergo cancer screening due to lack of knowledge, provider discrimination, and systemic barriers. - Healthcare providers often do not discuss screening recommendations with LGBTQ+ patients, leading to lower adherence. - Welcoming healthcare environments and nonjudgmental provider interactions improve screening rates. - Structural discrimination, including lack of LGBTQ+ data collection in cancer registries, limits research and policy interventions.	- Limited cancer screening guidelines specific to LGBTQ+ populations. - Lack of large-scale epidemiological studies on LGBTQ+ cancer screening behaviors. - Need for targeted interventions to increase screening adherence among LGBTQ+ individuals.
Haviland et al. (2021) [42]	Barriers to palliative care in sexual and gender minority (SGM) patients with cancer	USA	Scoping review	Review of 10 studies on LGBT+ patients in palliative cancer care	- LGBT+ individuals have higher cancer risks due to elevated tobacco, alcohol, and drug use. - Fear of discrimination and lack of provider education lead to late-stage cancer diagnoses, reducing palliative care effectiveness. - Barriers include discrimination, social isolation, disenfranchised grief, and lack of legal protections in advanced care planning. - Limited palliative care training on LGBT+ health disparities contributes to inadequate care.	- Insufficient research on LGBT+ patients’ specific needs in palliative oncology care. - Lack of culturally competent palliative care guidelines. - Minimal inclusion of sexual orientation and gender identity (SOGI) data in cancer research.
Heer et al. (2023) [43]	Participation, barriers, and facilitators of cancer screening among LGBTQ+ populations	International (USA, Canada, Australia, UK, Israel)	Literature review (50 studies analyzed)	LGBTQ+ individuals across various cancer screening studies	- Lesbian and bisexual women have lower participation in cervical cancer screening and mammography compared to heterosexual women. - Gay and bisexual men are more likely to participate in anal and colorectal cancer screening. - Transgender individuals have lower screening rates across all cancer types. - Key barriers include discrimination, lack of provider awareness, and fear of stigma. - The strongest facilitator is good communication with healthcare providers.	- Need for standardized cancer screening guidelines tailored to LGBTQ+ populations. - Lack of large-scale, representative data on screening behaviors. - Insufficient integration of LGBTQ+ cultural competence training in preventive healthcare.
Johnson et al. (2016) [44]	Quantitative and mixed analyses to identify factors that affect cervical cancer screening uptake among lesbian and bisexual women and transgender men	USA	Convergent parallel mixed-methods study	226 lesbian, bisexual, and queer women + transgender men (quantitative survey) + 20 in-depth interviews	- 73% were routine cervical screeners. - Routine screeners felt more welcomed in healthcare settings and were more likely to disclose their sexual orientation to providers. - Nonroutine screeners reported higher discrimination based on sexual orientation and gender expression. - Provider recommendation was a strong predictor of screening adherence.	- Lack of tailored interventions for increasing cervical cancer screening rates in LGBTQ+ populations. - Limited provider training on LGBTQ+ health disparities. - Need for inclusive healthcare environments and culturally competent care.
Joudeh et al. (2021) [45]	Barriers to accessing healthcare for sexual and gender minority (SGM) individuals in rural southern USA	USA	Qualitative study with intersectional approach	LGBTQ+ individuals from rural areas	- SGM individuals face stigma and discrimination from healthcare providers, leading to healthcare avoidance. - Economic and geographic barriers limit access to inclusive healthcare services. - Fear of being denied care or receiving inadequate treatment leads some to hide their identity when seeking medical assistance. - Support networks within LGBTQ+ communities help mitigate some of these barriers.	- Lack of LGBTQ+-inclusive training for healthcare providers in rural areas. - Insufficient policies to address structural discrimination in healthcare. - Need for intersectional approaches considering race, class, and economic disparities in LGBTQ+ health access.
Peters et al. (2021) [20]	Culturally safe, high-quality breast cancer screening for transgender people	Australia	Scoping review	Analysis of existing literature on breast cancer screening for transgender individuals	- Transgender people face barriers in accessing breast cancer screening, including discrimination, lack of provider knowledge, and inadequate screening guidelines. - There is limited research on breast cancer risk differences between transgender and cisgender individuals. - Gender-affirming treatments may influence breast cancer risk, but data are insufficient to determine screening recommendations. - There is a need for culturally safe and inclusive screening services.	- Lack of standardized breast cancer screening guidelines tailored for transgender individuals. - Limited large-scale studies on the impact of hormone therapy on breast cancer risk. - Need for training programs to improve healthcare providers’ competence in transgender healthcare.
Wang et al. (2025) [46]	Factors influencing oncology nurses’ culturally competent cancer care for LGBT individuals	Taiwan	Qualitative study	25 oncology nurses from different regions of Taiwan	- Oncology nurses’ attitudes, confidence, and beliefs significantly influence their willingness to provide culturally competent care to LGBT patients. - Interpersonal dynamics, including colleagues’ and managers’ attitudes, impact nurses’ comfort levels in providing inclusive care. - Organizational factors, such as hospital climate and availability of LGBT-related training, play a crucial role in shaping oncology nurses’ preparedness. - Societal and policy-level barriers, including lack of standardized LGBT patient intake forms and a generally conservative healthcare environment, hinder inclusive cancer care.	- Lack of systematic training programs on LGBT-inclusive cancer care for oncology nurses. - Insufficient institutional policies for collecting and using patients’ sexual orientation and gender identity (SOGI) data. - Need for interdisciplinary team approaches to improve LGBT cultural competence in oncology settings.
Sutter et al. (2020) [47]	Knowledge and attitudes of oncology advanced practice providers (APPs) toward sexual and gender minority (SGM) cancer patients	USA	Cross-sectional survey	78 oncology APPs (nurse practitioners, physician assistants) at an NCI-Designated Cancer Center	- APPs reported high comfort levels in treating SGM patients but had significant knowledge gaps regarding their specific healthcare needs. - Only 44.9% believed that knowing a patient’s sexual orientation is important for oncology care, while 67.9% acknowledged the importance of knowing gender identity. - 79.5% of respondents expressed interest in LGBTQ+ health education, yet only 47.4% supported mandatory training. - A significant decrease in self-reported confidence in LGBTQ+ health knowledge was observed after completing the survey.	- Lack of standardized educational programs addressing LGBTQ+ cancer care. - Limited provider training on inclusive practices in oncology settings. - Need for research on how knowledge gaps impact clinical outcomes for LGBTQ+ patients.
Roth et al. (2024) [48]	Experiences of hereditary cancer care among transgender and gender diverse (TGD) people	USA	Cross-sectional qualitative study	19 semi-structured interviews with TGD adults with hereditary cancer syndromes, family cancer histories, or chest cancer diagnoses	- TGD individuals experience significant barriers in hereditary cancer counseling, including misgendering, exclusion from decision-making, and lack of provider knowledge. - Cancer risk management decisions often intersect with gender-affirming care, such as mastectomy and hormone therapy. - Many patients experience distress due to gendered healthcare environments (e.g., “Women’s Imaging” centers). - Providers who use inclusive language and demonstrate allyship improve patient experiences.	- Lack of standardized hereditary cancer counseling guidelines for TGD individuals. - Insufficient provider training on gender-affirming care within genetic counseling. - Need for inclusive, gender-neutral medical environments to improve healthcare accessibility.
Kamen et al. (2018) [49]	LGBT cancer survivorship: identity, psychological distress, and barriers in care	USA	Mixed-method study (survey + literature review)	311 LGBT cancer survivors (online survey)	- LGBT cancer survivors face unique challenges in integrating their identity as cancer survivors with their sexual/gender identity. - Anxiety about coming out to multiple healthcare providers leads to delayed follow-up care. - Minority stress exacerbates post-treatment psychological distress, including anxiety and depression. - Family and relationship structures differ from heterosexual counterparts, often leading to exclusion of chosen family in care decisions.	- Lack of research on the long-term mental health outcomes of LGBT cancer survivors. - Insufficient guidelines for coordinating gender-affirming care with cancer survivorship. - Need for evidence-based interventions to reduce stigma and improve psychological support.
Kano et al. (2020) [50]	Addressing cancer disparities in sexual and gender minority (SGM) populations	USA	Policy and framework analysis	Review of research gaps, training deficits, and recommendations for national policy	- SGM individuals experience significant disparities in cancer prevention, treatment, and survivorship care due to provider knowledge gaps and structural barriers. - National Cancer Institute (NCI) funding for SGM cancer research is limited, leading to inadequate evidence-based guidelines. - Healthcare providers lack formal training on SGM-specific cancer care, leading to poor communication and clinical outcomes. - A National Action Plan is proposed to increase provider and researcher training on SGM cancer disparities.	- Limited SGM cancer research due to lack of funding and data collection. - Need for standardized cancer care guidelines for SGM populations. - Insufficient integration of SGM cultural competence into oncology education and practice.
Kano et al. (2023) [50]	Piloting the Sexual and Gender Minority Cancer Curricular Advances for Research and Education (SGM Cancer CARE) Workshop	USA	Educational intervention study	19 clinicians and researchers participating in the SGM Cancer CARE workshop	- Participants showed significant improvements in knowledge of SGM cancer disparities and research methodologies. - The workshop effectively increased awareness of culturally competent oncology care for SGM patients. - Virtual format facilitated accessibility but limited networking opportunities. - Need for expanding hybrid learning models to support inclusive cancer research training.	- Lack of standardized educational programs focusing on SGM cancer research. - Insufficient mentorship opportunities for early-career researchers in SGM oncology. - Need for long-term evaluation of the impact of such training programs on clinical practice and research outcomes.
Kerr et al. (2021) [51]	“I’m Not From Another Planet”: The Alienating Cancer Care Experiences of Trans and Gender-Diverse People	International	Qualitative study	Transgender and gender-diverse individuals receiving oncological treatment	- Trans and gender-diverse individuals experience significant alienation in oncology care settings due to stigma and discrimination. - Communication barriers with healthcare providers lead to a lack of trust and reluctance to seek care. - Lack of provider training results in inadequate management of hormone therapy interactions with cancer treatment. - Stereotypes and biases contribute to psychological distress and healthcare avoidance. - Support services for transgender patients are insufficient.	- Need for structured cultural competence training for healthcare providers. - Lack of research on the long-term impacts of gender-affirming treatments on cancer care. - Insufficient development of gender-inclusive healthcare policies in oncology.
Legere & MacDonnell (2016) [52]	Support for lesbian and bisexual women navigating reproductive cancer care.	Canada	Qualitative study	Lesbian and bisexual women facing reproductive cancer care.	- The study identifies meaningful support needs for lesbian and bisexual women in the context of reproductive cancer care in Canada, highlighting gaps in culturally competent care and challenges faced by these women in accessing appropriate healthcare services.	- The study suggests further research into the development of tailored support strategies and healthcare training programs for healthcare providers to improve care for lesbian and bisexual women in cancer treatment settings.
Levitt (2015) [53]	Clinical nursing care for transgender patients with cancer	USA	Clinical review	Analysis of barriers and best practices for transgender-inclusive oncology care	- Transgender individuals often avoid preventive and cancer care due to discrimination and systemic barriers. - Fear of stigmatization and past negative healthcare experiences contribute to delayed cancer diagnoses. - Limited research exists on malignancies related to hormone therapy, but available studies suggest they are rare. - Oncology nurses require specific education and training to provide culturally competent and inclusive care.	- Lack of large-scale research on cancer incidence and outcomes in transgender populations. - Need for structured oncology nursing education on transgender health issues. - Insufficient institutional policies ensuring transgender-inclusive oncology care.
Lombardo et al. (2022) [54]	Perceptions and barriers to cancer screening among sexual and gender minority (SGM) individuals	USA	Cross-sectional survey	422 SGM individuals (survey via social media)	- 65.4% of SGM individuals were uncertain about what cancer screening they needed. - Emotional distress was a significant barrier, with nearly 50% stating it prevented them from undergoing screenings. - Transgender individuals had the most significant knowledge gaps regarding screening and experienced higher distress. - Lack of provider training and structural barriers (e.g., misgendering, financial concerns) reduced screening adherence.	- Insufficient research on the impact of emotional distress on screening adherence. - Need for targeted interventions to improve awareness and accessibility of screenings for transgender individuals.
Radix & Maingi (2018) [55]	LGBT cultural competence in oncology nursing and healthcare	USA	Literature review	Review of existing literature on cultural competence training in oncology	- LGBT individuals experience significant health disparities, including lower cancer screening rates and worse health outcomes. - Minority stress, stigma, and discrimination contribute to avoidance of healthcare services. - Lack of cultural competence among oncology nurses negatively impacts the quality of care. - Training programs for nurses and healthcare providers improve health equity and patient satisfaction.	- Need for standardized LGBT cultural competence training in oncology nursing curricula. - Limited data on how training programs affect patient outcomes. - Insufficient policies to integrate LGBT cultural competence into routine cancer c
Mann-Barnes et al. (2023) [56]	Factors influencing HPV vaccination behavior among young men who have sex with men (YMSM)	USA	Cross-sectional survey	444 SM aged 18–27	- 75.79% of participants did not receive at least one dose of the HPV vaccine. - Healthcare provider recommendation was the strongest predictor of vaccine uptake (OR = 25.54, *p* < 0.001). - Stigma and homophobia increased the likelihood of vaccination uptake. - Higher numbers of sexual partners and condomless anal sex were associated with lower HPV vaccination rates.	- Need for targeted interventions to address stigma and improve provider recommendations. - Limited research on HPV vaccine accessibility in marginalized MSM communities. - Insufficient data on the long-term impact of HPV vaccination hesitancy in MSM populations.
Margolies (2014) [57]	Psychosocial needs of LGBT+ cancer patients	USA	Literature review and expert opinion	Analysis of barriers and challenges in cancer care for LGBT+ individuals	- LGBT+ individuals face increased cancer risks due to higher rates of smoking, alcohol use, and obesity. - Discrimination and secrecy contribute to poorer health outcomes, including lower screening rates and increased psychological distress. - LGBT+ cancer survivors experience additional challenges in treatment and survivorship due to inadequate provider knowledge and lack of inclusive policies. - A significant gap exists in the collection of sexual orientation and gender identity (SOGI) data in cancer registries.	- Limited research on long-term survivorship experiences of LGBT+ cancer patients. - Insufficient training programs for oncology nurses on LGBT+ health needs. - Need for standardized cancer screening guidelines tailored to LGBT+ populations.
McConkey & Holborn (2018) [58]	Lived experience of gay men with prostate cancer	Ireland	Phenomenological qualitative study	8 gay men diagnosed and treated for prostate cancer	- Gay men with prostate cancer experience unique challenges related to sexual dysfunction, identity, and support systems. - Many felt isolated due to a lack of tailored healthcare and support services. - Disclosure of sexual orientation to healthcare providers was influenced by past experiences of discrimination. - Lack of LGBT-inclusive cancer support groups and resources in Ireland.	- Insufficient healthcare provider education on LGBT-specific cancer survivorship needs. - Lack of prostate cancer rehabilitation programs tailored for gay men. - More research needed on the intersection of sexual identity and cancer treatment side effects.
Miller et al. (2020) [59]	Disclosure of sexual orientation and gender identity among Deaf LGBTQ patients	USA	Cross-sectional survey	313 af LGBTQ adults	- Cisgender women were significantly less likely to disclose their LGBTQ identity compared to cisgender men. - Acceptance from family and patient-centered communication with healthcare providers increased the likelihood of disclosure. - The presence of an ASL interpreter did not significantly impact disclosure decisions. - Healthcare providers need intersectional cultural competence to address the unique barriers faced by deaf LGBTQ individuals.	- Limited research on the intersection of disability and LGBTQ+ healthcare disparities. - Need for targeted training programs to improve provider communication skills for deaf LGBTQ patients. - Lack of data on health outcomes related to disclosure among deaf LGBTQ populations.
Nelson et al. (2023) [1]	Physician perceptions on cancer screening for LGBTQ+ patients	USA	Cross-sectional survey study	355 physicians across various specialties (oncology, radiology, internal medicine, family medicine)	- Only 28% of physicians received LGBTQ+-specific training. - 85% recognized that LGBTQ+ subpopulations have unique health concerns, but only 46% felt confident in understanding them. - Physicians with prior LGBTQ+ training were more likely to acknowledge the importance of knowing a patient’s sexual orientation and to be listed as LGBTQ+-friendly. - There was a lack of consensus on cancer screening guidelines for lesbian and transgender patients.	- Need for clearer national cancer screening guidelines for LGBTQ+ subpopulations. - More educational programs to enhance physician knowledge of LGBTQ+ health issues. - Research needed to determine the impact of physician training on cancer screening adherence among LGBTQ+ individuals.
Patel et al. (2024) [2]	Inclusive care practices for sexual and gender minority (SGM) patients with genitourinary cancer	USA	Literature review	Review of best practices for SGM-inclusive oncology care	- SGM patients face systemic barriers, provider biases, and lack of tailored healthcare resources, leading to poorer outcomes. - Use of organ-based language (e.g., “people with prostates” instead of “men with prostates”) improves inclusivity. - Gender-affirming care should be integrated with oncological treatment to address both physical and psychological needs. - Multidisciplinary approaches, including social work and onco-fertility services, enhance care quality.	- Limited research on genitourinary cancers in transgender and non-binary individuals. - Lack of standardization in collecting and using sexual orientation and gender identity (SOGI) data. - Need for training programs for oncologists to provide SGM-inclusive care.
Roznovjak et al. (2021) [3]	Breast cancer risk and screening in transgender individuals	USA	Literature review	Review of existing literature and cancer registry data on transgender breast cancer cases	- Transgender women on cross-sex hormone therapy have a higher risk of breast cancer compared to cisgender men but lower than cisgender women. - Transgender men have a higher breast cancer risk compared to cisgender men but lower than cisgender women. - Limited standardized guidelines exist for breast cancer screening in transgender populations. - Many transgender individuals face healthcare discrimination, leading to lower screening rates.	- Need for prospective studies to quantify breast cancer risk in transgender populations. - Lack of consensus on optimal screening recommendations. - Insufficient provider training on gender-affirming breast cancer care.
Rahman et al. (2019) [4]	Healthcare utilization and engagement among transgender and cisgender bisexual+ persons	USA	Cross-sectional survey	87 ciswomen, 34 transwomen, and 27 transmen, all identifying as bisexual, pansexual, or queer	- Bi+ transmen and transwomen were more likely to be uninsured or on government-sponsored insurance compared to ciswomen. - Transmen were less likely to have received a pelvic exam or Pap smear compared to ciswomen. - Transgender participants had significantly lower knowledge about HPV than ciswomen. - Bi+ trans individuals had lower comfort levels with healthcare providers, contributing to reduced engagement in preventive care.	- Need for targeted interventions to address healthcare disparities in bisexual and transgender individuals. - Insufficient research on the intersection of bisexuality and transgender healthcare needs. - More studies needed on effective strategies to improve preventive care utilization in these populations.
Scime (2019) [8]	Inequities in cancer care among transgender people	Canada	Literature review	Review of barriers, discrimination, and recommendations for transgender-inclusive oncology care	- Trans individuals face high levels of discrimination in oncology settings, leading to healthcare avoidance. - Social determinants of health, including economic disparities and lack of legal protections, impact cancer care access. - Cancer registries often fail to include gender identity data, limiting research on cancer outcomes in transgender populations. - Oncology nurses play a critical role in advocacy and policy change to create inclusive healthcare environments.	- Lack of large-scale prospective studies on cancer risk and outcomes in transgender individuals. - Need for transgender-specific cancer screening and treatment guidelines. - Insufficient training programs for oncology healthcare providers on transgender-inclusive care.
Seay et al. (2022) [10]	Effectiveness of LGBT cultural competency training for oncologists	USA	Randomized pragmatic trial	5000 oncologists invited, randomized into two training groups (COLORS training vs. general LGBT competency training)	- The COLORS training significantly improved oncologists’ knowledge, attitudes, and affirming clinical practices for LGBT+ patients. - Oncologists who completed the COLORS training reported increased confidence in discussing gender identity and sexual orientation with patients. - The study highlights the lack of standardized LGBT+ training in oncology education.	- Need for long-term assessment of training impact on patient outcomes. - Limited data on how training influences actual clinical practice changes. - Need for expansion of LGBT+ cultural competency training to other healthcare professionals beyond oncologists.
Schulz-Quach et al. (2024) [60]	Sexual and gender diversity in cancer care and survivorship	International	Literature review	Analysis of barriers and strategies in LGBTQ+ oncology care	- LGBTQ+ individuals face significant barriers in cancer care, including stigma, discrimination, and lack of culturally competent healthcare providers. - Psychological and social factors impact access to and quality of care. - Strategies such as cultural competency training, inclusive healthcare environments, and improved communication are essential for better cancer care. - Programs for raising awareness and reducing health disparities should be widely implemented.	- Need for more research on long-term cancer survivorship in LGBTQ+ populations. - Limited studies on the intersection of LGBTQ+ identity and palliative oncology care. - Insufficient integration of inclusive practices in mainstream cancer care guidelines.
Tamargo et al. (2017) [11]	Knowledge, attitudes, and practice behaviors of oncology providers toward LGBTQ+ patients	USA	Cross-sectional survey	388 oncology providers, 108 completed the survey (27.8% response rate), and 36 specialized in LGBTQ-prevalent cancers	- Less than 50% of surveyed providers correctly answered knowledge questions about LGBTQ-specific health risks. - 91.7% felt comfortable treating LGBTQ patients, but only 47.2% believed they were well informed on LGBTQ health needs. - 77.8% agreed that more education on LGBTQ health should be included in medical training. - Most providers assumed patients were heterosexual upon first encounter (72.2%).	- Need for standardized LGBTQ-specific training programs in oncology. - Lack of provider knowledge about cancer disparities in LGBTQ populations. - Insufficient research on how provider attitudes impact cancer care outcomes for LGBTQ individuals.
Taylor et al. (2019) [14]	Access to cancer knowledge and its mobilization among LGBQ/T patients	Canada, USA	Qualitative study	81 LGBQ/T individuals diagnosed and treated for breast and/or gynecological cancer	- LGBQ/T patients face structural and systemic barriers in accessing cancer care. - Most rely on online sources for cancer-related knowledge due to lack of culturally competent healthcare providers. - Many feel uncomfortable disclosing their gender/sexual identity to providers, impacting trust and quality of care. - Online cancer support spaces are often cisnormative and heteronormative, excluding LGBQ/T needs.	- Lack of research on the role of online information in cancer decision-making for LGBQ/T patients. - Need for more inclusive cancer education materials and culturally competent oncology care. - Insufficient training for providers to address LGBQ/T cancer health disparities.
Burkhalter et al. (2016) [12]	The National LGBT Cancer Action Plan	USA, UK, Canada	White paper (expert consensus)	56 invited experts in LGBT cancer research, clinical care, policy, and survivorship	- LGBT individuals face significant disparities in cancer risk, screening, treatment, and survivorship due to stigma and discrimination. - There is a lack of sexual orientation and gender identity (SOGI) data collection in national cancer registries, hindering research and policy efforts. - Oncology providers often lack training in LGBT-inclusive care. - The Action Plan proposes 16 recommendations to address disparities, including improving data collection, funding LGBT cancer research, and increasing provider education.	- Need for standardized SOGI data collection in cancer surveillance programs. - Limited research on cancer risk factors, treatment outcomes, and survivorship in LGBT populations. - Insufficient funding and institutional support for LGBT-focused oncology research.
Ziegler et al. (2024) [9]	Cancer screening and prevention in the transgender and gender diverse (TGD) population	Canada, Australia	Discussion paper based on literature review and guidelines	Review of barriers, strategies, and role of Advanced Practice Nurses (APNs) in TGD cancer care	- TGD individuals face systemic barriers to cancer screening, including discrimination, lack of provider knowledge, and inadequate gender-affirming care. - Cancer prevention and screening services are often designed for cisgender individuals, creating access barriers for TGD populations. - APNs can play a key role in advocating for gender-affirming cancer screening guidelines and improving culturally competent care. - There is a need for tailored interventions and research on the long-term cancer risks for TGD individuals, particularly those undergoing hormone therapy.	- Need for more research on cancer prevalence and risk factors specific to TGD populations. - Limited evidence on the effectiveness of gender-affirming screening guidelines. - Insufficient training programs for APNs and other healthcare providers on TGD cancer care.

## Data Availability

No new data were created or analyzed in this study.
